# The Possible Role of Extravillous Trophoblast-Derived Exosomes on the Uterine Spiral Arterial Remodeling under Both Normal and Pathological Conditions

**DOI:** 10.1155/2014/693157

**Published:** 2014-09-14

**Authors:** Carlos Salomon, Sarah W. Yee, Murray D. Mitchell, Gregory E. Rice

**Affiliations:** UQ Centre for Clinical Research, Centre for Clinical Diagnostics, Royal Brisbane and Women's Hospital, Herston, Brisbane, QLD 4029, Australia

## Abstract

A tenet of contemporary obstetrics is that events that compromise placentation increase the risk of complications of pregnancy and contribute to poor pregnancy outcome. In particular, conditions that affect the invasion of placental cells and remodeling of uterine spiral arteries compromise placental function and the subsequent development of the fetus. Extravillous trophoblast cells (EVTs) proliferate and migrate from the cytotrophoblast in the anchoring villi of the placenta and invade the maternal decidua and myometrium. These cells are localised with uterine uterine spiral arteries and are thought to induce vascular remodeling. A newly identified pathway by which EVTs may regulate vascular remodeling within the uterus is via the release of exosomes. Trophoblast cells release exosomes that mediate aspects of cell-to-cell communication. The aim of this brief commentary is to review the putative role of exosomes released from extravillous trophoblast cells in uterine spiral artery remodeling and, in particular, their role in the aetiology of preeclampsia. Placental exosomes may engage in local cell-to-cell communication between the cell constituents of the placenta and contiguous maternal tissues and/or distal interactions, involving the release of placental exosomes into biological fluids and their transport to a remote site of action.

## 1. Introduction

A successful outcome to pregnancy is critically dependent upon events that affect implantation and early development of the placenta [1]. After implantation, trophoblast cells (CTs) that arise from blastocyst proliferate and differentiate into syncytiotrophoblasts (STs) and EVTs [2]. During first trimester, the placenta develops under low oxygen tension (~3% O_2_) that, in part, is maintained by intravascular EVTs occluding uterine spiral arteries and preventing maternal blood from perfusing the placenta intervillous space. Remodeling of the uterine spiral arteries (SpA) into low resistance, high capacity vessels begins as EVTs invade the decidua during first trimester [3]. When EVTs “plugs” are lost between 9 and 11 weeks of gestation, maternal blood flows through the modified vessels to deliver nutrients and oxygen to support fetal growth and development [4]. EVTs continue to invade into the myometrium and remodel the SpA until mid-second trimester [5–8]. While the mechanisms by which EVTs remodel SpA remain to be fully elucidated, available data are consistent with the hypothesis that EVTs directly interact with vascular smooth muscle cells of uterine spiral arteries and affect their loss.

Over the past five years, our understanding of how cells communicate with each other, in health and disease, has undergone a paradigm shift with the recognition of the role of exosomes in intercellular signalling [9, 10]. Exosomes are small (40–100 nm), very stable [11], and lipid bilayer nanovesicles that are formed by the inward budding of multivesicular bodies. Although we know little about the mechanism by which exosomal packaging occurs, they contain a diverse array of signalling molecules and are released from the parent cell following the exocytotic fusion of multivesicular bodies with the cell membrane [12]. In this brief commentary, we develop the working hypothesis that exosomal signalling plays a critical role in normal placentation and that disruption of exosomal pathways (and in particular the release of exosomes from EVTs) plays a key role in the pathogenesis of complications of pregnancy, including preeclampsia.

## 2. EVTs and Uterine Spiral Artery Remodeling

Remodeling of uterine spiral arteries by EVTs is fundamental for effective placentation and perfusion of the intervillous space. Approximately 100–150 uterine spiral arteries are transformed during placental development [13]. The main role of these vessels is to transport maternal blood to the placenta to support the growth and development of the fetus. This is achieved by converting arteries from high resistance low flow to high flow low resistance arteries [14]. The diameter of uterine spiral arteries during early pregnancy is 200 *μ*m [8]. After remodeling, arteries have an average luminal size of 2 mm [15]. Dysfunctional remodeling of uterine spiral arteries is associated with complications of pregnancy, such as preeclampsia.

The principal placental cell type involved in uterine spiral artery remodeling is the EVT. EVTs invasion occurs through the interstitial pathway and endovascular pathway [16]. Interstitial EVTs migrate through the uterine stroma and endovascular EVTs through the distal end of the uterine spiral arteries [17]. By the eighth week of pregnancy, interstitial EVTs invade the decidua [18].

After week 10, endovascular EVTs cells invade decidua segment of uterine spiral arteries from the cytotrophoblastic shell [19]. Invasion by EVTs causes temporary artery plugging which decreases maternal blood flow that protects the fetus from oxidative stress [20]. When the plug disintegrates, endovascular EVT will further invade into the myometrium from week 14. These trophoblast cells will interact with the endothelium of the vessel and deposit fibrinoid material [5].

The initial steps of uterine spiral artery remodeling consist of vessel dilatation, vascular smooth muscle cell separation, endothelial cell swelling, EVTs infiltration, and fibrinoid deposition [17]. Vascular smooth muscle cells migrate or undergo apoptosis and are replaced by fibrinoid material, in which EVTs cells embed. The precise cellular mechanisms by which vascular smooth muscle cells are lost from the uterine spiral arteries are not known. Possible mechanisms include migration, apoptosis, and inhibition of proliferation and dedifferentiation [16]. Apoptosis of vascular smooth muscle cell is a process that occurs in normal pregnancy to maintain vessel homeostasis [21]. Vascular smooth muscle cell migration into decidual stroma and into the lumen of vessels is associated with several cytokines, growth factors, and breaking down of extracellular matrix [21].

## 3. Microenvironmental Factors

The functions of EVTs are affected by intrauterine microenvironmental factors, including oxygen tension and inflammatory mediators.

### 3.1. Oxygen Tension

Placentation is an oxygen sensitive process. The events that occur from the time of implantation to maternal perfusion of the placenta are influenced and directed by site-specific oxygen tensions [22]. An oxygen gradient exists between the placenta and endometrium during the first trimester. At the time of embryo implantation, the intrauterine oxygen tension is 3% [23] while the decidua and myometrium oxygen tension is 8–12% [24]. This standing oxygen gradient is thought to promote and direct the invasion of EVTs into the decidua and myometrium where they remodel maternal uterine spiral arteries [25]. Intraluminal EVTs occlude uterine spiral arterioles to maintain a low oxygen tension environment that is requisite for normal early placental and fetal development. Towards the end of the first trimester, low resistance, high capacity flow is achieved by the loss of intraluminal trophoblast plugs and the placental intravillous space is perfused with maternal blood, thus establishing effective maternofetal exchange. Dysfunctional placentation is associated with a failure to remodel uterine spiral arteries, abnormal placental perfusion, and oxygenation (similar to ischemia-reperfusion injury). After vascular remodeling of the SpA, the oxygen tension increase in the placenta [26]. These developmental changes in oxygen tension are thought to be an obligate regulator of cell function and phenotype. When perturbed, placentation and the subsequent perfusion of the placenta may be compromised. Activation of HIF-1*α* and inflammatory signalling pathways have been implicated in this process.

### 3.2. Inflammatory Mediators

Inflammation has a main role in supporting tissue homeostasis; indeed normal healthy pregnancy is characterised as a controlled, mild proinflammatory state [27]. The expression of inflammatory mediators is required to achieve a successful pregnancy that involves a series of intercellular interactions, particularly, at the site of implantation and placentation [28]. The inflammatory microenvironment is regulated by a balance between release of proinflammatory and anti-inflammatory cytokines [29]. These molecules have a critical and essential role in the maternal adaptation to the requirement of the different stages of gestation [30]. Complications of pregnancies such as fetal growth restriction and preeclampsia are frequently related to irregular maternal inflammation.

Tumor necrosis factor-α (TNF-α) is a proinflammatory cytokine produced by different cells, such as fibroblast, macrophages, vascular cells, uterine NK cells, and placental cells that can promote trophoblasts growth and invasion [31–34]. It has been demonstrated that TNF-*α* have a key role in trophoblast migration into maternal decidua and spiral arterial remodeling [35, 36]; however, the mechanisms involving the transformation of uterine spiral arteries by EVTs cells have not been fully understood. TNF-α is a pleiotropic cytokine that has been found to be involved in many activities in preeclampsia [30]. In this instance, we believe that placental hypoxia is a consequence of arterial remodeling failure influenced by proinflammatory conditions in preeclampsia.

TNF-α was first detected in placental supernatants and amniotic fluid [37, 38]. Expression of TNF-α in placenta changes during pregnancy and is responsive to changes in the extracellular milieu [39] suggesting that TNF-α has a specific function in developmental processes [40]. Expression of TNF-α mRNA in the first trimester of pregnancy has been found in all cell types belonging to the trophoblastic lineage. TNF-α expression, however, decreases in invasive cells at later stages of pregnancy [41]. TNF-α activates proapoptotic factors as well as antiapoptotic factors to maintain a microenvironment for successful arterial remodeling. It has been reported that trophoblast differentiation could be regulated by TNF-α [42].

The aetiological antecedents of preeclampsia are thought to be aberrant maternal-fetal immune tolerance that reduced trophoblast invasion. Recent studies have shown that immune maladaptation and overt activation of maternal immune system may be responsible in the pathogenesis of preeclampsia [43]. In the past decades, serum levels of TNF-α had elevated and increased expressions of TNF-α and TNF receptors were found in leukocytes and placenta of women with preeclampsia [40]. This rise can occur as early as 11–13 weeks of pregnancy, much earlier than detectable clinical manifestations [44]. TNF-α may inhibit EVT migration in first trimester placenta via activated macrophages. In early onset of preeclampsia, findings on TNF-α and interleukin-2 (an anti-inflammatory cytokine) suggested that there is an imbalance of proinflammatory and anti-inflammatory cytokines ratio [45]. Toll-like receptor which is the main danger signalling pathway involved in the pathophysiology of preeclampsia increases the production of TNF-α [46]. Another study performed by Hamai et al., [47] has shown an increase of TNF-α in early pregnancy of preeclampsia. In asymptomatic patients (patients who later developed preeclampsia in the second trimester), the level of TNF-*α* in the first trimester was 2-fold higher compared to healthy controls [47]. On the other hand, other authors have demonstrated that level of TNF-α increased significantly in women diagnosed with preeclampsia compared with healthy control [48–50].

Preeclampsia is characterised with reduced uteroplacental perfusion and incomplete uterine spiral arterial remodeling. Moreover, a high level of TNF-*α* has been found in plasma from patients with preeclampsia; however, the role of TNF-*α* in the failure of spiral artery remodeling and the mechanisms involved in this phenomenon still are not fully elucidated. In this regard, it has been established recently that small vesicles released by many cell types including human placental cells contain a membrane bound form of TNF-*α* [51]. Recent studies highlight the putative utility of tissue-specific nanovesicles (e.g., exosomes) in the diagnosis of disease onset and treatment monitoring [9, 52–56]. To date, there is a paucity of data defining changes in the release, role, and diagnostic utility of placenta-derived exosomes in pregnancies complicated by preeclampsia.

## 4. Exosomes: Definition and Characteristics

**Figure 1:**
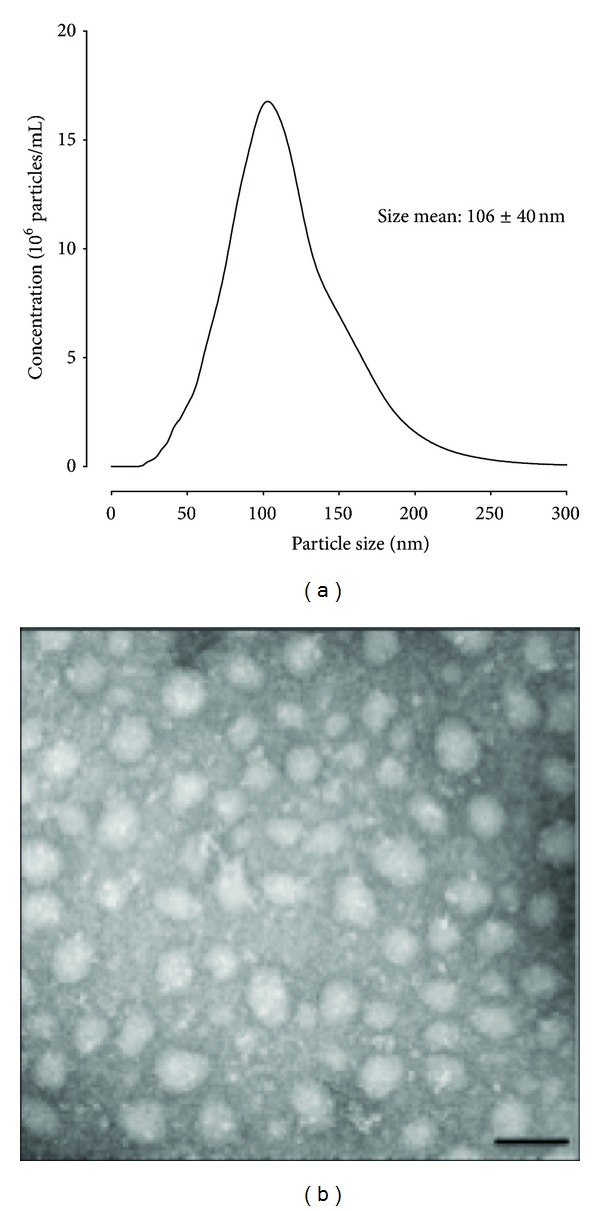
Placental exosomes characterisation. Exosomes vesicles were isolated from placental cells by differential and buoyant density centrifugation and purified using a sucrose continuous gradient as previously described [65]. (a) Representative graph size distribution of the exosome samples using a nanoparticle tracking analysis (NanoSight NS500). (b) Representative electron micrograph of enriched exosomes population. In (b), scale bar is 200 nm.

Exosomes are small (40–100 nm) and very stable membrane vesicles that are released when late endosomal bodies fuse with the cell membrane [57, 58]. Exosomes found in cell cultures and body fluids indicate that they can be released from different types of cells [59]. Exosomes are characterised by a buoyant density of 1.13–1.19 g/mL, an endosomal origin, and enrichment of late endosomal membrane markers (including Tsg101, CD63, CD9, and CD81), are released into extracellular compartments [60], and are identified in most biological fluids examined [61, 62]. Exosomes are generated by the inward budding of late endosomal structures, the multivesicular bodies (MVB). Moreover, the participation of Rab GTPases in the secretion of exosomes has been proposed [63]. Although we know little about the mechanism by which packaging occurs, exosomes contain a diverse array of signalling molecules and are released from the parent cell following the exocytosis fusion of multivesicular bodies with the cell membrane [12]. Signalling molecules, including miRNA; mRNA; and cytoplasmic proteins, are packaged into exosomes. Exosomal signalling occurs when released exosomes fuse with target cells and deploy their contents to alter cell function. In pathological pregnancies, exosomes secreted from the placenta may be involved in adaptive responses and different biological processes such as metabolism, development, cellular adhesion, and immune response of the mother and fetus. We have isolated and characterised exosomes released from placental cells and have demonstrated that trophoblast cells release exosomes that are bioactive and can regulate the biological function on cell target [58, 64, 65]. A representative standard size distribution graph and electron microscope image of the exosome samples isolated from placental cells are shown in Figure 1.

### 4.1. Exosomes and Cell-to-Cell Communication

Exosomes interact with target cells via multiple pathways, by directly activating target cell membrane receptors; by modifying the extracellular* milieu* of the target cell; and by fusing with the cell membrane and releasing their molecular cargo into the target cell [66]. Recently, it has been demonstrated that cells internalise exosomes through lipid raft-mediated endocytosis involving caveolin-1 protein and ERK1/2-heat shock protein 27 signaling in this process [67]. Their molecular cargo is cell specific [68], regulated by tissue physiology and cellular function, and fundamental to their bioactivity.

Exosomes may be assembled and secreted in response to instructions received from neighbouring cells, from distant tissues, or in response to local environmental factors (e.g., oxygen tension). Their molecular cargos, including mRNA [69], miRNA [68, 69], proteins [65], lipids [70], and membrane receptors, are transferred to adjacent cells and/or distal cells via biofluid transport (e.g., in blood, lymph, saliva, or ascites).

Currently, we have only a limited understanding of the role that exosomal signaling plays in normal physiology and pathophysiology and, in particular, in reproductive biology. This provides us with exciting opportunities to establish the role of exosomes in disease pathology and to advance diagnosis and treatment of clinically significant conditions.

Placental cells release exosomes* in vitro* and* in vivo* and have been identified in maternal blood [64, 71, 72]. They contain placenta-specific protein and miRNA and, as such, may be differentiated from maternally derived exosomes [53, 73]. The concentration of exosomes has been reported to increase in association with some complications of pregnancy (e.g., preeclampsia [72]). In this regard, complications of pregnancy are associated with a proinflammatory state (e.g., high TNF-α concentrations) and also with failure in the SpA remodeling where EVTs have been demonstrated to have an important role. Our group has isolated and characterised exosomes released from placental cells and has demonstrated that (i) first trimester cytotrophoblast (CT) cells release exosomes^CD63+,CD9+,CD81+,PLAP+^ *in vitro *[65]; (ii) CT-exosome release and protein content are regulated by oxygen tension; and (iii) CT exosomes induce extravillous cytotrophoblast cell invasion and proliferation in a time- and dose-dependent manner [65]. In addition to direct effects on target cells, exosomes from nongestational tissues have been reported to remodel the extracellular matrix (ECM) surrounding target cells (i.e., cell fusion-independent effects). We have identified serine proteases (e.g., HtrA 4, which is expressed by CTs and syncytiotrophoblast (ST); present in maternal plasma; and increased in association with PE [74]) as well as metalloproteases (e.g., MMP 2, MMP 9, and MMP 12) in CT exosomes [65].

Recently, it has been proposed that MMP 12 secreted by trophoblast cells induces disruption of uterine vascular smooth muscle cell architecture favouring extravillous trophoblast invasion [75, 76]. The activity and capacity of trophoblast-derived exosomes to directly bind and remodel ECM in a cell fusion-independent manner have yet to be established. Exosomal remodeling of ECM may participate not only in cytotrophoblasts-extravillous trophoblasts interactions but also in the extravillous trophoblast-endothelial cells and extravillous trophoblast-vascular smooth muscle cell interactions. As we know that exosomes protect their content, we hypothesised that EVT-derived exosomes interact with vascular cells (i.e., smooth muscle and endothelial cells), delivering their specific cargo (e.g., MMPs) and contributing to the SpA remodeling.

### 4.2. Oxygen Tension Can Regulate the Effect of Placental Exosomes

Recently, we reported that changes in oxygen tension also regulate placental exosome release, content, and bioactivity [58, 65]. Hypoxia (1% O_2_) increases the release of exosomes from CTs incubated* in vitro* when compared to CTs incubated under 3% or 8% O_2_. The protein content of these “hypoxic” exosomes is also altered with increased enrichment of HIF-1*α* and IL-8 signalling molecules. In addition, the ability of these exosomes to induce cell migration is significantly enhanced. Oxygen tension also regulates the responsiveness of target cells to exosomes. This phenomenon has been demonstrated in other cell types (e.g., cancer cells), where exosomes content reflects the oxygenation status of cells [84]. These data provide new insights and understanding into how oxygen tension regulates cell function and, in particular, the role of oxygen tension in regulating exosomal signalling in the placenta. Our preliminary studies identify oxygen-dependent changes in the protein content of CT exosomes; however, effects on miRNA mediators remain to be established. Using nongestational tissue cell lines (epithelial ovarian cancer cells), we have also identified cell-specific packaging of miRNA into exosomes [68]. We will use this approach to identify cell- and treatment-specific effects on miRNA packaging into trophoblast exosomes. Human placenta and placental-derived exosomes express the chromosome 19 miRNA cluster (C19MC), which is regulated selectively by hypoxic stress [85]. Moreover, it has also been demonstrated that trophoblast cells utilise exosomes for the transfer of specific and unique miRNA (from cluster C19MC) to other cells (e.g., maternal and fetal cells) and confer them with viral resistance against infections [78]. Placental-derived exosomes under both normal and pathological conditions could perform a main role in the maternal adaptation to pregnancy (e.g., uterine vascular adaptation to pregnancy).

### 4.3. Exosomes Regulate Cell Migration on Cell Target

Exosomes mediate cell-to-cell communication and induce different effects on target cells depending on the cell origin and exosome content (e.g., miRNA and proteins). The function of placental-derived exosomes during normal or pathological pregnancy remains to be established. Several studies support the hypothesis that placental exosomes (i.e., release from cytotrophoblast, extravillous trophoblasts, and syncytiotrophoblast) are capable of promoting cell migration (Table 1). In addition, this phenomenon not only is restrictive to placental exosomes but also has been demonstrated in nonplacental exosomes [82]. We have previously reported that exosomes released from cytotrophoblast cells primary culture contain biologically active proteins [65] that can interact with the maternal endothelium and regulate their function (e.g., migration and angiogenesis). Furthermore, the release of exosome from placental mesenchymal stem cells and cytotrophoblast cells is regulated by the oxygen tension [58, 65]. Exosomes isolated from Swan 71 cells (trophoblastic cell lines) promote monocytes migration and increased the production of proinflammatory cytokines from these cells [86]. Primary human trophoblast cells are resistant to viral infection (e.g., human cytomegalovirus) and can transfer their viral resistance to nonplacental cells (i.e., endothelial cells) through exosomes, an effect completely abolished by sonication [78], highlighting that the exosome integrity is critical to mediate their effects on cell target.

We have recently demonstrated that exosomes isolated from peripheral plasma were biologically active, as assessed by their ability to increase endothelial cell migration* in vitro. *Moreover, the bioactivity of exosomes was greatest during the first trimester and gradually declined with advancing gestational age. These results suggest that, in normal pregnancy, exosomes isolated from plasma of pregnant healthy women in the first trimester may play a role in regulating the endothelium response to maternal adaptation to pregnancy. Exosomes are sensitive to environmental milieu (e.g., oxygen tension), changing their bioactivity and content; we propose that, under physiological conditions (e.g., normal pregnancy), placental exosomes promote vascular cell migration from the uterine spiral arteries; however, under pathological conditions (e.g., proinflammatory state and preeclampsia), the bioactivity of placental exosomes is reduced.

### 4.4. Preeclampsia Is Associated with Increased Release of Placenta-Derived Vesicles

Preeclampsia (PE) is a leading cause of maternal and fetal morbidity and mortality with an incidence rate of 3–5% of all pregnancies [88, 89]. One of the first events associated with development of PE is the failure in remodeling the uterine maternal arteries completely and consequently the inadequate placental blood flow. While the precise etiology of PE remains largely unknown, physiological, environmental, and immunological risk factors have been identified [89]. The hypothesis that trophoblast-derived vesicles and debris shed into maternal circulation promotes an inflammatory vascular response and causes endothelial damage that is correlated with the pathophysiology of PE that has been proposed by Redman et al.'s group [90]. The placental syncytiotrophoblast secretes a wide range of vesicles, including micro- and nanovesicles into the maternal circulation during normal pregnancy [64]. Using a flow cytometry approach and syncytiotrophoblast-specific antiplacental alkaline phosphatase (PLAP), significantly greater levels of placental-derived vesicles were found in both peripheral and uterine venous plasma from women with preeclampsia compared to normal pregnant women [91]. Moreover, similar results were observed using a dual placental perfusion system in placentae from preeclampsia pregnancy [92]. In contrast, a recent study using nanoparticles tracking analysis reported high level of placental-derived vesicles in pregnant women compared with nonpregnant women, without difference in the number of syncytiotrophoblast extracellular vesicles between normal pregnant women and plasma from patients with preeclampsia [93]. To our knowledge, wide variation between results can be attributed to methodological differences, while flow cytometer is still inadequate to detect single vesicles with size less 300 nm (without polystyrene beads) and the expression of PLAP is reduced in syncytiotrophoblast-derived vesicles (including micro- and nanovesicles) obtained from perfused placental from preeclamptic pregnancies [92].

The concentration of placenta-derived exosomes vesicles is also increased with the advancing gestational age [64]. The molecular composition and biological effects of these nanovesicles are determined by their cellular origin. Thus, events that impact on early trophoblast cell invasion and their interaction with maternal cells (including oxygen tension and glucose and fatty acid concentrations) may contribute to or predispose to complication of pregnancies [64]. It has been demonstrated that exosomal protein content is different in women with preeclampsia [94]. Moreover, the specific syncytiotrophoblast protein, syncytin-2, is markedly downregulated in exosomes derived from placenta of pregnant women with preeclampsia compared to healthy control (normal pregnancies) [95].

In contrast, high levels of syncytiotrophoblast-derived vesicles were found in plasma from women with early-onset preeclampsia [96]. Since trophoblast invasion and insufficient uterine vascular remodeling occur in early-onset preeclampsia, we, therefore, propose that the release and composition (i.e., exosomal proteins) of placenta-derived exosomes are altered in pregnancies that subsequently develop complications (e.g., preeclampsia) and that placental cell exosomes derived from abnormal pregnancies differentially affect vascular smooth muscle cell function.

## 5. Summary

**Figure 2:**
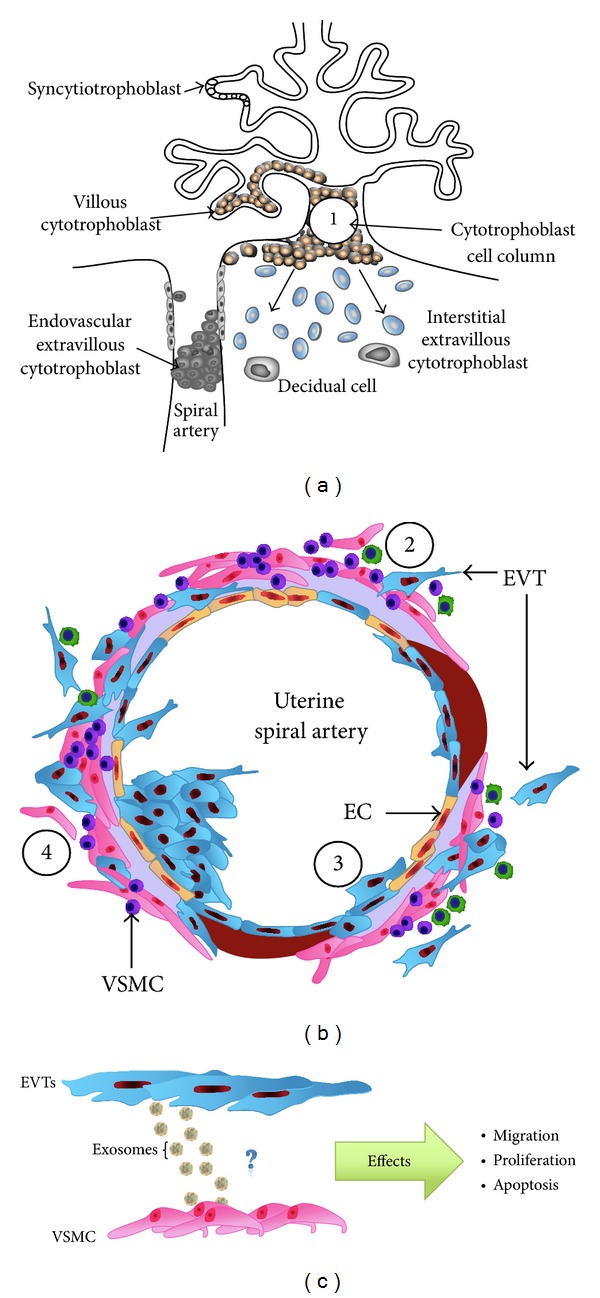
A hypothesis on the effect of EVTs-derived exosomes on SpA remodeling. Complications of pregnancy are thought to be clinical manifestations of a common developmental lesion inadequate invasion by extravillous trophoblast cells with a consequent failure to remodel the maternal uterine spiral arteries. EVTs migrate from the cytotrophoblast-anchoring villi ((a) [1]) of the placenta and invade the maternal decidua and myometrium. These cells are localised with uterine spiral arteries (b) and are thought to induce vascular remodeling (i.e., extracellular matrix remodeling [2]); the loss of vascular endothelial [3]; and smooth muscle [4] cells by apoptosis or migration out of the vessel wall. We propose that EVTs-derived exosome has participation on the SpA remodeling, specificity affecting process as migration, proliferation, and apoptosis of VSMC (c). In (b), cartoon is modified from Cartwright et al., 2010 [87].

Uterine spiral arterial remodeling is an important physiological change during early pregnancy. EVTs migrate into maternal decidua and myometrium and interact with endothelial and vascular smooth muscle cells in uterine spiral arteries. Conversion of these arteries is associated with the loss of both endothelial cells and vascular smooth muscle cells from the vessel wall by apoptosis and/or migration out of the vessel. In this regard, communication between EVTs and vascular smooth muscle cells appears to be essential for successful arterial remodeling. The effect of exosomes released from EVTs on endothelial cells and vascular smooth muscle cells has not been established. We propose that in complicated pregnancies (e.g., preeclampsia), proinflammatory microenvironment regulates the release and bioactivity of EVT-derived exosomes. In normal pregnancy, EVT-derived exosomes may promote vascular smooth muscle cell migration favoring the spiral uterine arterial remodeling; however, high concentration of proinflammatory cytokines (e.g., TNF-*α*) may inhibit the effect of exosomes on vascular smooth muscle cell migration, triggering failure in arterial remodeling and stimulating the emergence of preeclampsia (Figure 2).

## Figures and Tables

**Table 1:** Effects of exosomes vesicles on cell target.

Vesicles source	Isolation methods	Cell target	Biological function	Effect	References
Cytotrophoblast cells	UT + sucrose continuous gradient	EVT (HTR-8/Svneo)	Invasion and proliferation	Promote	[65]
pMSC	UT + 30% sucrose cushion	hPMEC	Migration and proliferation	Promote	[58]
Maternal plasma	UT + sucrose continuous gradient	HUVEC	Migration	Promote	[64]
Trophoblast (Swan 71)	UT	Monocytes	Migration	Promote	[54]
Chorionic villi explant	UT + sucrose continuous gradient	Jurkat T cells and PBMC	Apoptosis	Promote	[77]
Trophoblast cells	UT + 30% sucrose cushion	HUVEC	Viral infection	Resistance	[78]
Human macrophages	UT + sucrose continuous gradient	Endothelial cell	Migration	Decrease	[79]
CML cells	UT + 30% sucrose cushion	HUVEC	Migration	Promote	[80]
Dendritic cells	UT + 30% sucrose cushion	PBMC	Migration	Promote	[81]
Pancreatic adenocarcinoma cells	UT + sucrose continuous gradient	Endothelial cells	Migration	Promote	[82]
HUVEC	UT	SMCs	miRNAs expression	Transfer miRNAs	[83]

UT: ultracentrifugation (>100,000 ×g); EVT: extravillous trophoblast; pMSC: placental mesenchymal stem cells; hPMEC: human placental microvascular endothelial cells; HUVEC: human umbilical vein endothelial cells; PBMC: peripheral blood mononuclear cells; CML: chronic myelogenous leukemia; SMCs: smooth muscle cells.

## References

[1] Lappas M, Rice GE (2009). Transcriptional regulation of the processes of human labour and delivery. *Placenta*.

[2] Gude NM, Roberts CT, Kalionis B, King RG (2004). Growth and function of the normal human placenta. *Thrombosis Research*.

[3] Kam EPY, Gardner L, Loke YW, King A (1999). The role of trophoblast in the physiological change in decidual spiral arteries. *Human Reproduction*.

[4] Pijnenborg R, Ball E, Bulmer JN, Hanssens M, Robson SC, Vercruysse L (2006). In vivo analysis of trophoblast cell invasion in the human. *Methods in molecular medicine.*.

[5] Pijnenborg R, Bland JM, Robertson WB, Brosens I (1983). Uteroplacental arterial changes related to interstitial trophoblast migration in early human pregnancy. *Placenta*.

[6] Blackburn CA, Keelan JA, Taylor RS, North RA (2003). Maternal serum activin A is not elevated before preeclampsia in women who are at high risk. *American Journal of Obstetrics and Gynecology*.

[7] American Diabetes Association (2012). Diagnosis and classification of diabetes mellitus. *Diabetes Care*.

[8] Boyd JD, Hamilton WJ (1970). *The Human Placenta*.

[9] Simpson RJ, Jensen SS, Lim JWE (2008). Proteomic profiling of exosomes: current perspectives. *Proteomics*.

[10] Simpson RJ, Lim JWE, Moritz RL, Mathivanan S (2009). Exosomes: proteomic insights and diagnostic potential. *Expert Review of Proteomics*.

[11] Kalra H, Adda CG, Liem M (2013). Comparative proteomics evaluation of plasma exosome isolation techniques and assessment of the stability of exosomes in normal human blood plasma. *Proteomics*.

[12] Harding CV, Heuser JE, Stahl PD (2013). Exosomes: looking back three decades and into the future. *Journal of Cell Biology*.

[13] Lyall F (2005). Priming and remodelling of human placental bed spiral arteries during pregnancy: a Review. *Placenta*.

[14] Burton GJ, Woods AW, Jauniaux E, Kingdom JCP (2009). Rheological and physiological consequences of conversion of the maternal spiral arteries for uteroplacental blood flow during human pregnancy. *Placenta*.

[15] Benirschke K, Kaufmann P (2000). *Pathology of the Human Placenta*.

[16] Whitley GSJ, Cartwright JE (2010). Cellular and molecular regulation of spiral artery remodelling: lessons from the cardiovascular field. *Placenta*.

[17] Pijnenborg R, Vercruysse L, Hanssens M (2006). The uterine spiral arteries in human pregnancy: facts and controversies. *Placenta*.

[18] Pijnenborg R, Bland JM, Robertson WB, Dixon G, Brosens I (1981). The pattern of interstitial trophoblastic invasion of the myometrium in early human pregnancy. *Placenta*.

[19] Pijnenborg R, Dixon G, Robertson WB, Brosens I (1980). Trophoblastic invasion of human decidua from 8 to 18 weeks of pregnancy. *Placenta*.

[20] Burton GJ, Jauniaux E, Watson AL (1999). Maternal arterial connections to the placental intervillous space during the first trimester of human pregnancy: the Boyd collection revisited. *The American Journal of Obstetrics and Gynecology*.

[21] Bulmer JN, Innes BA, Levey J, Robson SC, Lash GE (2012). The role of vascular smooth muscle cell apoptosis and migration during uterine spiral artery remodeling in normal human pregnancy. *The FASEB Journal*.

[22] Burton GJ, Jauniaux E, Charnock-Jones DS (2010). The influence of the intrauterine environment on human placental development. *International Journal of Developmental Biology*.

[23] Rodesch F, Simon P, Donner C, Jauniaux E (1992). Oxygen measurements in endometrial and trophoblastic tissues during early pregnancy. *Obstetrics and Gynecology*.

[24] Jauniaux E, Gulbis B, Burton GJ (2003). Physiological implications of the materno-fetal oxygen gradient in human early pregnancy. *Reproductive BioMedicine Online*.

[25] Lyall F (2006). Mechanisms regulating cytotrophoblast invasion in normal pregnancy and pre-eclampsia. *Australian and New Zealand Journal of Obstetrics and Gynaecology*.

[26] Wang Y, Zhao S (2010). *Vascular Biology of the Placenta*.

[27] Borzychowski AM, Sargent IL, Redman CWG (2006). Inflammation and pre-eclampsia. *Seminars in Fetal and Neonatal Medicine*.

[28] Redman CWG, Sargent IL (2004). Preeclampsia and the systemic inflammatory response. *Seminars in Nephrology*.

[29] LaMarca BD, Ryan MJ, Gilbert JS, Murphy SR, Granger JP (2007). Inflammatory cytokines in the pathophysiology of hypertension during preeclampsia. *Current Hypertension Reports*.

[30] Raghupathy R (2013). Cytokines as key players in the pathophysiology of preeclampsia. *Medical Principles and Practice*.

[31] Chen H-L, Yang Y, Hu XL, Yelavarthi KK, Fishback JL, Hunt JS (1991). Tumor necrosis factor alpha mRNA and protein are present in human placental and uterine cells at early and late stages of gestation. *The American Journal of Pathology*.

[32] Vince G, Shorter S, Starkey P (1992). Localization of tumour necrosis factor production in cells at the materno/fetal interface in human pregnancy. *Clinical and Experimental Immunology*.

[33] Philippeaux M-M, Piguet PF (1993). Expression of tumor necrosis factor-α and its mRNA in the endometrial mucosa during the menstrual cycle. *The American Journal of Pathology*.

[34] Jokhi PP, King A, Sharkey AM, Smith SK, Loke YW (1994). Screening for cytokine messenger ribonucleic acids in purified human decidual lymphocyte populations by the reverse-transcriptase polymerase chain reaction. *Journal of Immunology*.

[35] Bauer S, Pollheimer J, Hartmann J, Husslein P, Aplin JD, Knöfler M (2004). Tumor necrosis factor-α inhibits trophoblast migration through elevation of plasminogen activator inhibitor-1 in first-trimester villous explant cultures. *Journal of Clinical Endocrinology and Metabolism*.

[36] Goetze S, Xi X-P, Kawano Y (1999). TNF-α-induced migration of vascular smooth muscle cells is MAPK dependent. *Hypertension*.

[37] Laham N, Brennecke SP, Bendtzen K, Rice GE (1994). Tumour necrosis factor α during human pregnancy and labour: maternal plasma and amniotic fluid concentrations and release from intrauterine tissues. *European Journal of Endocrinology*.

[38] Laham N, Van Dunné F, Abraham LJ (1997). Tumor necrosis factor-*β* in human pregnancy and labor. *Journal of Reproductive Immunology*.

[39] Coughlan MT, Oliva K, Georgiou HM, Permezel JMH, Rice GE (2001). Glucose-induced release of tumour necrosis factor-alpha from human placental and adipose tissues in gestational diabetes mellitus. *Diabetic Medicine*.

[40] Haider S, Knöfler M (2009). Human tumour necrosis factor: physiological and pathological roles in placenta and endometrium. *Placenta*.

[41] Pijnenborg R, McLaughlin PJ, Vercruysse L (1998). Immunolocalization of tumour necrosis factor-α (TNF-α) in the placental bed of normotensive and hypertensive human pregnancies. *Placenta*.

[42] Knöfler M, Mösl B, Bauer S, Griesinger G, Husslein P (2000). TNF -α/TNFRI in primary and immortalized first trimester cytotrophoblasts. *Placenta*.

[43] Rusterholz C, Hahn S, Holzgreve W (2007). Role of placentally produced inflammatory and regulatory cytokines in pregnancy and the etiology of preeclampsia. *Seminars in Immunopathology*.

[44] Williams MA, Farrand A, Mittendorf R (1999). Maternal second trimester serum tumor necrosis factor-α-soluble receptor p55 (sTNFp55) and subsequent risk of preeclampsia. *American Journal of Epidemiology*.

[45] Xie F, Hu Y, Turvey SE (2010). Toll-like receptors 2 and 4 and the cryopyrin inflammasome in normal pregnancy and pre-eclampsia. *BJOG*.

[46] Xie F, Turvey SE, Williams MA, Mor G, von Dadelszen P (2010). Toll-like receptor signaling and pre-eclampsia. *The American Journal of Reproductive Immunology*.

[47] Hamai Y, Fujii T, Yamashita T (1997). Evidence for an elevation in serum interleukin-2 and tumor necrosis factor-α levels before the clinical manifestations of preeclampsia. *American Journal of Reproductive Immunology*.

[48] Kalantar F, Rajaei S, Heidari AB (2013). Serum levels of tumor necrosis factor-alpha, interleukin-15 and interleukin-10 in patients with pre-eclampsia in comparison with normotensive pregnant women. *Iranian Journal of Nursing and Midwifery Research*.

[49] Lau SY, Guild S-J, Barrett CJ (2013). Tumor necrosis factor-alpha, interleukin-6, and interleukin-10 levels are altered in preeclampsia: a systematic review and meta-analysis. *The American Journal of Reproductive Immunology*.

[50] Xie C, Yao MZ, Liu JB, Xiong LK (2011). A meta-analysis of tumor necrosis factor-alpha, interleukin-6, and interleukin-10 in preeclampsia. *Cytokine*.

[51] Zhang HG, Liu C, Su K (2006). A membrane form of TNF-alpha presented by exosomes delays T cell activation-induced cell death. *Journal of Immunology*.

[52] Chen Y, Ge W, Xu L (2012). miR-200b is involved in intestinal fibrosis of Crohn’s disease. *International Journal of Molecular Medicine*.

[53] Atay S, Gercel-Taylor C, Kesimer M, Taylor DD (2011). Morphologic and proteomic characterization of exosomes released by cultured extravillous trophoblast cells. *Experimental Cell Research*.

[54] Atay S, Gercel-Taylor C, Suttles J, Mor G, Taylor DD (2011). Trophoblast-derived exosomes mediate monocyte recruitment and differentiation. *American Journal of Reproductive Immunology*.

[55] Armitage J, Poston L, Taylor P (2007). Developmental origins of obesity and the metabolic syndrome: the role of maternal obesity. *Frontiers of Hormone Research*.

[56] Taylor DD, Gerçel-Taylor C (2005). Tumour-derived exosomes and their role in cancer-associated T-cell signalling defects. *British Journal of Cancer*.

[57] Théry C (2011). Exosomes: secreted vesicles and intercellular communications. *F1000 Biology Reports*.

[58] Salomon C, Ryan J, Sobrevia L (2013). Exosomal signaling during hypoxia mediates microvascular endothelial cell migration and vasculogenesis. *PLoS ONE*.

[59] Vlassov AV, Magdaleno S, Setterquist R, Conrad R (2012). Exosomes: current knowledge of their composition, biological functions, and diagnostic and therapeutic potentials. *Biochimica et Biophysica Acta: General Subjects*.

[60] Simons M, Raposo G (2009). Exosomes—vesicular carriers for intercellular communication. *Current Opinion in Cell Biology*.

[61] Lässer C, Alikhani VS, Ekström K (2011). Human saliva, plasma and breast milk exosomes contain RNA: uptake by macrophages. *Journal of Translational Medicine*.

[62] Keller S, Ridinger J, Rupp A-K, Janssen JWG, Altevogt P (2011). Body fluid derived exosomes as a novel template for clinical diagnostics. *Journal of Translational Medicine*.

[63] Ostrowski M, Carmo NB, Krumeich S (2010). Rab27a and Rab27b control different steps of the exosome secretion pathway. *Nature Cell Biology*.

[64] Salomon C, Torres MJ, Kobayashi M (2014). A gestational profile of placental exosomes in maternal plasma and their effects on endothelial cell migration. *PLoS ONE*.

[65] Salomon C, Kobayashi M, Ashman K, Sobrevia L, Mitchell MD, Rice GE (2013). Hypoxia-induced changes in the bioactivity of cytotrophoblast-derived exosomes. *PLoS ONE*.

[66] Pegtel DM, Cosmopoulos K, Thorley-Lawson DA (2010). Functional delivery of viral miRNAs via exosomes. *Proceedings of the National Academy of Sciences of the United States of America*.

[67] Svensson KJ, Christianson HC, Wittrup A (2013). Exosome uptake depends on ERK1/2-heat shock protein 27 signaling and lipid raft-mediated endocytosis negatively regulated by caveolin-1. *The Journal of Biological Chemistry*.

[68] Kobayashi M (2014). Ovarian cancer cell invasiveness is associated with discordant exosomal sequestration of Let-7 miRNA and miR-200. *Journal of Translational Medicine*.

[69] Valadi H (2007). Exosomes contain a selective number of mRNA and microRNA. *Allergy*.

[70] Record M, Carayon K, Poirot M, Silvente-Poirot S (2014). Exosomes as new vesicular lipid transporters involved in cell-cell communication and various pathophysiologies. *Biochimica et Biophysica Acta—Molecular and Cell Biology of Lipids*.

[71] Sabapatha A, Gercel-taylor C, Taylor DD (2006). Specific isolation of placenta-derived exosomes from the circulation of pregnant women and their immunoregulatory consequences. *American Journal of Reproductive Immunology*.

[72] Redman CWG, Sargent IL (2008). Circulating microparticles in normal pregnancy and pre-eclampsia. *Placenta*.

[73] Luo S-S, Ishibashi O, Ishikawa G (2009). Human villous trophoblasts express and secrete placenta-specific microRNAs into maternal circulation via exosomes. *Biology of Reproduction*.

[74] Inagaki A, Nishizawa H, Ota S (2012). Upregulation of HtrA4 in the placentas of patients with severe pre-eclampsia. *Placenta*.

[75] Harris LK, Smith SD, Keogh RJ (2010). Trophoblast- and vascular smooth muscle cell-derived MMP-12 mediates elastolysis during uterine spiral artery remodeling. *The American Journal of Pathology*.

[76] Harris LK (2011). IFPA Gabor Than Award lecture: transformation of the spiral arteries in human pregnancy: key events in the remodelling timeline. *Placenta*.

[77] Stenqvist AC, Nagaeva O, Baranov V, Mincheva-Nilsson L (2013). Exosomes secreted by human placenta carry functional Fas ligand and TRAIL molecules and convey apoptosis in activated immune cells, suggesting exosome-mediated immune privilege of the fetus. *The Journal of Immunology*.

[78] Delorme-Axford E, Donker RB, Mouillet JF (2013). Human placental trophoblasts confer viral resistance to recipient cells. *Proceedings of the National Academy of Sciences of the United States of America*.

[79] Lee HD, Kim YH, Kim DS (2014). Exosomes derived from human macrophages suppress endothelial cell migration by controlling integrin trafficking. *European Journal of Immunology*.

[80] Taverna S, Flugy A, Saieva L (2012). Role of exosomes released by chronic myelogenous leukemia cells in angiogenesis. *International Journal of Cancer*.

[81] Esser J, Gehrmann U, D'Alexandri FL (2010). Exosomes from human macrophages and dendritic cells contain enzymes for leukotriene biosynthesis and promote granulocyte migration. *Journal of Allergy and Clinical Immunology*.

[82] Nazarenko I, Rana S, Baumann A (2010). Cell surface tetraspanin Tspan8 contributes to molecular pathways of exosome-induced endothelial cell activation. *Cancer Research*.

[83] Hergenreider E, Heydt S, Tréguer K (2012). Atheroprotective communication between endothelial cells and smooth muscle cells through miRNAs. *Nature Cell Biology*.

[84] Kucharzewska P, Christianson HC, Welch JE (2013). Exosomes reflect the hypoxic status of glioma cells and mediate hypoxia-dependent activation of vascular cells during tumor development. *Proceedings of the National Academy of Sciences of the United States of America*.

[85] Donker RB, Mouillet JF, Chu T (2012). The expression profile of C19MC microRNAs in primary human trophoblast cells and exosomes. *Molecular Human Reproduction*.

[86] Atay S, Gercel-Taylor C, Taylor DD (2011). Human trophoblast-derived exosomal fibronectin induces pro-inflammatory IL-1*β* production by macrophages. *American Journal of Reproductive Immunology*.

[87] Cartwright JE, Fraser R, Leslie K, Wallace AE, James JL (2010). Remodelling at the maternal-fetal interface: relevance to human pregnancy disorders. *Reproduction*.

[88] Steegers EA, von Dadelszen P, Duvekot JJ, Pijnenborg R (2010). Pre-eclampsia. *The Lancet*.

[89] Lorquet S, Pequeux C, Munaut C, Foidart J-M (2010). Aetiology and physiopathology of preeclampsia and related forms. *Acta Clinica Belgica*.

[90] Redman CWG, Tannetta DS, Dragovic RA (2012). Review: does size matter? Placental debris and the pathophysiology of pre-eclampsia. *Placenta*.

[91] Knight M, Redman CWG, Linton EA, Sargent IL (1998). Shedding of syncytiotrophoblast microvilli into the maternal circulation in pre-eclamptic pregnancies. *British Journal of Obstetrics and Gynaecology*.

[92] Tannetta DS, Dragovic RA, Gardiner C, Redman CW, Sargent IL (2013). Characterisation of syncytiotrophoblast vesicles in normal pregnancy and pre-eclampsia: expression of Flt-1 and endoglin. *PLoS ONE*.

[93] Dragovic RA, Southcombe JH, Tannetta DS, Redman CW, Sargent IL (2013). Multicolor flow cytometry and nanoparticle tracking analysis of extracellular vesicles in the plasma of normal pregnant and pre-eclamptic women. *Biology of Reproduction*.

[94] Redman CWG, Tannetta DS, Dragovic RA (2012). Review: does size matter? Placental debris and the pathophysiology of pre-eclampsia. *Placenta*.

[95] Vargas A, Zhou S, Ethier-Chiasson M (2014). Syncytin proteins incorporated in placenta exosomes are important for cell uptake and show variation in abundance in serum exosomes from patients with preeclampsia. *FASEB Journal*.

[96] Goswamia D, Tannetta DS, Magee LA (2006). Excess syncytiotrophoblast microparticle shedding is a feature of early-onset pre-eclampsia, but not normotensive intrauterine growth restriction. *Placenta*.

